# Mammalian Anatomy

**Published:** 1898-12

**Authors:** 


**Mammalian Anatomy.** By HORACE JAYNE, M.D. Part I.—*The Skeleton of the Cat.* Pp. xix., 816. Philadelphia: J. B. Lippincott Company. 1898.

The first volume of this work, a magnificent book, splendidly printed and lavishly illustrated, fills a gap which has long been recognised.

The way in which the subject has been treated deserves every commendation. The author states that its object is to pave the way for the study of human anatomy; and to this end each bone is represented by a beautifully drawn figure, which puts to shame the figures in most works on comparative anatomy, and which represents, where present, the outlines of muscular attachment in a way which is familiar to students of human anatomy. In addition, each figure is compared with one representing a corresponding bone in man; and as nomenclature has been reduced to a common denominator, the task of the reader is reduced to a minimum, for he knows exactly where he is. If the book had nothing else to recommend it, this last point would be sufficient.

The student who has worked with this book as his guide proceeds directly with the study of human anatomy, and the names he meets with are quite familiar to him; and as the cat is an easily-obtained animal for dissection, the student of human anatomy who wishes to make himself acquainted with comparative anatomy may use this book as a key, and he will save himself endless trouble. A very valuable description of the teeth is given, and as it is accompanied by excellent drawings, the utmost use can be made of it.

We should, however, like to criticise the description of the cervical vertebræ as given here, more especially with reference to the means given of recognising individual vertebræ. All teachers of human anatomy expect their pupils to recognise individual bones by absolute not relative characteristics; and when we are told, as we are here, that a vertebra may be recognised by its being larger than another, or wider or longer, such a description must be, on the face of it, of little value. If it is to be of any value, any description for purposes of recognition must enable us to distinguish a given isolated bone. Now it is as easy to recognise an isolated third, fourth, fifth, sixth, or seventh cervical vertebra in a cat by absolute characteristics as it is in man, perhaps easier; and so it is in the majority of mammals, certainly in the carnivora and rodentia. We do not mention this in a spirit of captious criticism, but from a feeling that such a very excellent work, professing to lead up to human anatomy, should be as complete and accurate as possible.

On the whole the book is a very valuable contribution to comparative anatomy, and is to be heartily recommended.

